# Exolysin Shapes the Virulence of *Pseudomonas aeruginosa* Clonal Outliers

**DOI:** 10.3390/toxins9110364

**Published:** 2017-11-09

**Authors:** Emeline Reboud, Pauline Basso, Antoine P. Maillard, Philippe Huber, Ina Attrée

**Affiliations:** CNRS-ERL5261, INSERM, U1036, CEA, Bacterial Pathogenesis and Cellular Responses, Biosciences and Biotechnology Institute of Grenoble, University Grenoble Alpes, 17 rue des Martyrs, CEA-Grenoble, 38054 Grenoble, France; emeline.reboud@gmail.com (E.R.); pauline.basso@gmail.com (P.B.); antoine.maillard@cea.fr (A.P.M.)

**Keywords:** two-partner secretion, pore-forming toxin, type IV pili, cell junctions, ADAM10, inflammation, PA7-like strains, ExlA

## Abstract

Bacterial toxins are important weapons of toxicogenic pathogens. Depending on their origin, structure and targets, they show diverse mechanisms of action and effects on eukaryotic cells. Exolysin is a secreted 170 kDa pore-forming toxin employed by clonal outliers of *Pseudomonas aeruginosa* providing to some strains a hyper-virulent behaviour. This group of strains lacks the major virulence factor used by classical strains, the Type III secretion system. Here, we review the structural features of the toxin, the mechanism of its secretion and the effects of the pore formation on eukaryotic cells.

## 1. Introduction

Protein toxins are main weapons of bacterial pathogens. Most Gram-negative pathogens, including the major human opportunist *Pseudomonas aeruginosa*, secrete toxins in their surrounding milieu or inject toxic effectors directly into host-cell cytoplasm.

*P. aeruginosa* is a ubiquitous bacillus that thrives in most moisture environments and colonizes different hosts from plants to insects, and to mammals [1,2,3]. *P. aeruginosa* is particularly well adapted to human hosts and is capable of provoking infections of different tissues and organs, such as urinary tract, lungs, eyes and damaged skin. It is often associated with nosocomial infections in Intensive Care Units and affects elderly patients with underlying chronic diseases, as well as patients with cystic fibrosis where it is the primary cause of morbidity and mortality [4]. Whole-genome analysis approaches have clustered environmental and clinical isolates of *P. aeruginosa* into three major groups [5,6], represented by reference strains PAO1, PA14 and PA7 [7,8,9,10]. While the majority of world-wide collected samples populates the two first groups (“PAO1” and “PA14”), the third group (“PA7”) harbours strains qualified as clonal outliers based on their sequence and genome divergence [5,6,11,12,13].

The main difference between clonal outliers and the rest of the population is the way those bacteria exert their cytotoxicity and virulence toward mammalian hosts. The “classical” strains belonging to “PAO1” and “PA14” groups possess the well-studied virulence determinant Type III Secretion System (T3SS). T3SS is a molecular syringe that allows the export of bacterial proteins and their injection directly into the host cell across three membranes [14,15]. The T3SS machinery is encoded by a four-operon chromosomal locus of ~25 kb containing genes for structural and regulatory proteins (reviewed in [14]). Four toxin-effectors are transported by the T3SS machinery, two of them, ExoS and ExoU, being mutually exclusive in a majority of clinical strains [16,17]. In contrast, clonal outliers of the “PA7” group are devoid of a T3SS machinery and all T3SS toxins [7,18]. Instead, they employ a secreted toxin of 170 kDa, named Exolysin (ExlA), to establish infection [19]. Exolysin has been first detected by non-targeted proteomics in the secreted medium of a highly cytotoxic strain CLJ1, isolated from a patient with chronic obstructive pulmonary disease (COPD) accompanied by signs of haemorrhage in lungs [19]. The recombinant expression of *exlBA* was found to be sufficient to cause fatal outcome in a mouse model of acute pneumonia and both genes are necessary for cytotoxicity toward eukaryotic cells. The *exlBA* locus encoding the toxin is absent in classical strains of *P. aeruginosa*, and is located between genes corresponding to PA0874 and PA0873 in the reference strain PAO1. This genetic organization is conserved in all clonal outliers sequenced to date. Bioinformatics analysis performed on all genomes available in the *Pseudomonas* database [10] revealed the presence of *exlBA* genes in several *Pseudomonas* species, including *P. putida*, *P. entomophila*, *P. protegens* and *P. fluorescens* [20]. Here we bring insights regarding the structural and functional features of this novel toxin acquired by some multi-resistant *P. aeruginosa* strains.

## 2. Exolysin Belongs to a Family of Two-Partner-Secretion Pore-Forming Toxins

**Figure 1:**
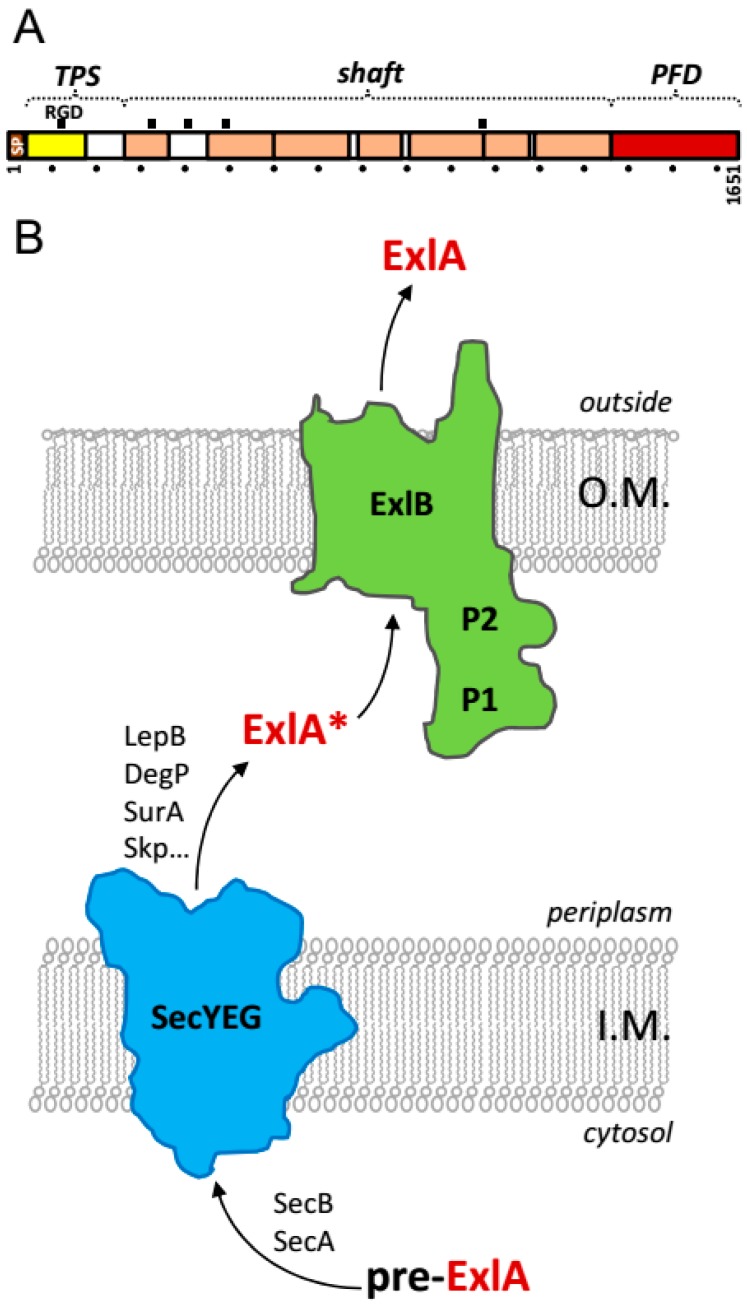
(**A**) Linear representation of ExlA showing the signal peptide (SP) and Pfam annotations [25] corresponding to the TPS domain (yellow) and Fil_Haemagg_2 repeats (salmon). The C-terminal pore-forming domain (PFD, red) made no hit in Pfam. A ruler is displayed below the diagram with a mark of every 100 residues. The five RGD motifs and the putative domains of ExlA are shown on top. (**B**) Schematic representation of the ExlA secretion pathway. ExlB and ExlA precursors are targeted to the periplasm via the general secretion pathway by recognition of their N-terminal signal peptides. With analogy to the ShlBA TPS, ExlB inserts into the outer-membrane where it forms the translocation pore to which the non-functional periplasmic intermediate ExlA* is targeted via the interaction between its TPS domain and ExlB POTRA domains P1 and P2. Some chaperones usually involved in protein secretion are mentioned (SecB, SurA, Skp, DegP).

The *exlA* gene encodes a 1651 residue-long polypeptide that bears successive sequence signatures typical of (i) a signal peptide recognized by the general Sec machinery, (ii) a so-called Two-Partner Secretion (TPS) domain and (iii) several filamentous hemagglutinin (FH) repeats (Figure 1A). The absence of any known sequence signature beyond residue 1364 defines a ~280 residue-long C-terminal domain that was proved necessary for ExlA activity toward eukaryotic cells (see below) [19,21]. Upstream from *exlA* is *exlB* that encodes a 60 kDa outer membrane (OM) protein channel of the Omp85 superfamily [22]. Proteins of this superfamily possess a C-terminal 16-stranded membrane-embedded β-barrel downstream a variable number of POlypeptide TRansport Associated (POTRA) domains, of which ExlB has two. Together, *P. aeruginosa* ExlB and ExlA make a secretion system that belongs to a system that was named TPS systems, such as to account for the minimal set of proteins specifically involved in secretion, i.e., the effector and its cognate transporter [23]. This dominant picture shall not occlude instances where additional dedicated partners may modify, neutralize or proteolyse the substrate [22], although none of this seems to apply to ExlA which was detected as a single ~170 kDa band after SDS-PAGE analysis of bacterial culture supernatants. Some founding members of that group are well characterized homologs of ExlBA, like *S. marcescens* ShlBA and *B. pertussis* FhaCB. With analogy to these well-studied TPS systems [24], ExlA secretion is predicted to occur in two steps: (i) the 34 residue-long, cleavable signal peptide at the N-terminus of ExlA targets the precursor to the general secretion pathway, which delivers ExlA to the periplasm after signal peptide processing and (ii) mature ExlA gets translocated across the outer membrane through ExlB (Figure 1B). Targeting of ExlA to ExlB across the periplasm probably requires the TPS domain of ExlA to interact with ExlB POTRA domains 1 and 2, making them indispensable for ExlA secretion [21].

While ExlA was originally detected in the bacterial culture supernatant, it is not clear whether the secreted form is the active one, i.e., its poor solubility could severely restrain diffusion of the toxin in the milieu, perhaps tethering it to the envelope. Indeed, the fact that ExlA-mediated virulence requires close contact between bacteria and target cell also suggests that the diffusion of secreted ExlA is confined around *P. aeruginosa*. This is in agreement with the limited diffusion reported for *S. marcescens* ShlA [26], with whom ExlA shares 34% overall identity.

**Figure 2:**
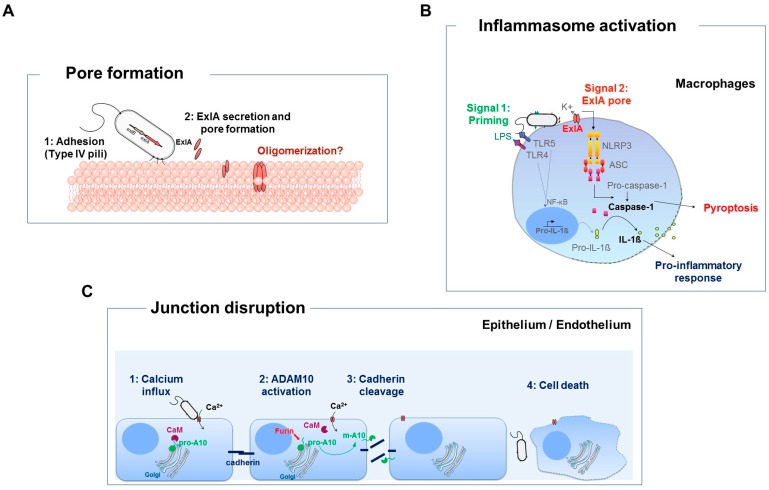
Effects of pore formation on eukaryotic cells. (**A**) When ExlA is secreted close to host cells, it can insert into the plasma membrane. Whether ExlA oligomerizes to form a pore is unknown. The type IV pili facilitate ExlA pore formation by promoting bacterial adhesion to the host cell, and thus its delivery near the membrane. (**B**) ExlA-secreting bacteria induce inflammasome activation and pyroptosis of macrophages (adapted from [20]). The priming signal is provided by LPS-TLR-4 or flagellum-TLR-5 interaction, leading to NF-κB nuclear translocation and synthesis of pro-IL-1β. The second signal is provided by ExlA pore formation inducing K^+^ efflux. Alteration in K^+^ cytosolic concentration elicits NLRP3/ASC inflammasome activation, which in turn activates caspase-1. Caspase-1 has two major effects, (i) cleavage of pro-IL-1β into mature IL-1β and (ii) the rupture of the macrophage plasma membrane and the massive release if IL-1β, which promotes pro-inflammatory response. (**C**) ExlA induces the cleavage of the intercellular adhesive proteins E- and VE-cadherins in epithelial and endothelial cells, respectively, by diverting a natural mechanism of cadherin shedding of the host (adapted from [32]). In resting conditions, the metalloprotease ADAM10 is maintained inactive (pro-A10) within a complex with calmodulin (CaM), a protein with high affinity for Ca^2+^. ExlA pore formation induces a massive entry of Ca^2+^ in the cytosol. The CaM-Ca^2+^ association liberates pro-A10, which is available for cleavage by furin and for export to the plasma membrane. In turn, ADAM10 (A10) cleaves the cadherins, inducing junction disruption. Later on, Ca^2+^ entry also induces necrotic cell death by unknown mechanisms.

ExlA belongs to a family of cytolysins/hemolysins, whose activity has been best characterized for ShlA. ShlA is responsible for the cell-bound hemolytic phenotype of *S. marcescens* [27]. ShlB and ShlA have been a major model in the early characterization of TPS. With bacterial cells and urea-extracts to palliate the minute-long half-life of the native toxin (hence “cell-bound”), in vitro hemolysis experiments allowed to establish that ShlA was secreted in two steps, the periplasmic form ShlA* being inactive, and hemolytic activity being acquired by ShlA concomitantly with outer-membrane translocation [28]. Strikingly, periplasmic ShlA obtained from *shlB* mutants of *S. marcescens* could be activated by either ShlB or secreted ShlA truncation constructs comprising the TPS domain [26]. A similar mechanism of activation has been documented for HpmA [29] and likely operates for ExlA. Active ShlA inserts in erythrocyte membranes where it adopts a new conformation [30] and makes 1-to-3 nm wide pores [27,31], similarly to what was found for ExlA (Figure 2A).

## 3. Affinity of Exolysin to Membranes

Pore forming toxins (PFT) classically dock to a receptor as they reach a target membrane, be it a protein, a sugar moiety or a lipid head group. After binding, the PFTs oligomerize then form a pore into the plasma membrane [33]. The measure of haemoglobin release from red blood cells in presence of osmoprotectants of different sizes allowed to suggest that once inserted, ExlA assembles into ~1.6 nm-wide pores [21]. The requirement for lipids in ExlA-mediated pore formation was investigated in vitro using liposomes. It was found that ExlA requires phosphatidylserine (PS) to provoke liposome leakage, probably involving electrostatic and hydrophobic interactions with this anionic phospholipid [21]. Likewise, a dependence on lipid head group ionic charge was shown for the membrane insertion of T3SS translocon proteins PopB and PopD [34]. In the case of ShlA, liposome leakage was observed in presence of PS and enhanced in presence of phosphatidylethanolamine (PE) [35].

Neither ExlA harvested from *P. aeruginosa* supernatant nor purified non-native ExlA showed lytic activity on eukaryotic cells. Moreover, only bacteria in contact with eukaryotic cells were able to exert ExlA-dependent cytolysis. Using a cellular based screen for bacterial factors that may promote ExlA pore-forming activity, type IV pili were identified to facilitate ExlA-mediated cytotoxicity. Therefore, pili may support ExlBA function by bringing the bacteria close to the host cell membrane. This proximity would enhance a local concentration of ExlA near the membrane and trigger pore formation [21] (Figure 2A) highlighting the originality of this TPS pore-forming toxin. The question whether or not ExlA/ShlA family of pore forming toxins use a specific cellular receptor for docking to the host membranes, and if yes, the identity of this receptor, remains open.

## 4. Virulence of *P. aeruginosa* Strains Secreting Exolysin

The current cohort of ExlA-positive strains (ExlA+) includes isolates from various pathological conditions (ear and urinary tract infections, burns, abscesses, bacteremia, cystic fibrosis, pneumonia, COPD) or from the environment (plant and pond water) [18]. Laboratory assays for some general virulence features showed that only a small number of these strains exert LasB-type proteolytic activity or production of HCN, two virulence factors produced by most classical strains (“PAO1” and “PA14”). Similar to other *P. aeruginosa* strains, ExlA+ strains display a great diversity in their capacity to swim, swarm or twitch. All eukaryotic cell types tested (epithelial, endothelial, fibroblastic, myeloid) were found permissive to ExlA-cytotoxicity, suggesting that ExlA+ bacteria may affect various organs and tissues in vivo. The role of ExlA in the virulence of PA7-like strains was provided by two experiments: (i) strain toxicity levels on cells were correlated with the amount of secreted ExlA, and (ii) *exlA* mutation abrogated cytoxicity. Moreover, survival curves in mice showed gradient in in vivo toxicity, which was not directly predictable from in vitro cytotoxicity assays on cell lines, suggesting that the interaction with the immune components is variable between strains and plays a pivotal role in global pathogenesis [18]. In a pulmonary infection model in mice, the most virulent strain, CLJ1, induces a mortality significantly higher than that of the PAO1 strain expressing the T3SS and ExoS, ExoT and ExoY effectors [19]. The histological examination of CLJ1-infected lungs indicated the presence of erythrocytes in the alveoli space, in addition to infiltration of neutrophils, thus reproducing the hemorrhagic symptoms of the patient. Transmission electron microscopy showed that CLJ1 provoked striking lesions in the alveolar wall. In particular, the tissues were disorganized and some pneumocytes and endothelial cells were necrotic with their cytoplasmic contents spilled in alveoli and capillaries [36]. These types of lesions were not observed in lungs infected with the T3SS-positive strain PAO1. ExlA clearly endows the bacteria with the ability to proliferate in lungs and to disseminate from lungs to other organs: liver, spleen, kidney and brain. Therefore, the rupture of the alveolo-capillary barrier induced by ExlA opens the passage to the blood compartment allowing bacterial dissemination. However, when the blood was directly infected in a mouse bacteremia model, all *P. aeruginosa* strains were promptly eliminated, independently of the secretion of ExlA or T3SS-toxins, and even more rapidly for the ExlA+ strain CLJ1. These results show that the bacterial survival in blood and their ability to resist to the immune system depends mostly on bacterial genetic background rather than on the particular secreted toxin(s) [36].

The ShlA toxin from *S. marcescens* induces similar damage on lungs, i.e., hemorrhagic pneumonia, recruitment of neutrophils and rapid lung dysfunction followed by mouse death [37]. Some other PFTs induce dramatic damage on lungs, either by direct tissue damage or by inflammatory side effects; the two mechanisms being often dependent on each other. For example, *Streptococcus pneumoniae*, PLY toxin causes the destruction of lung tissue, mediated by the induction of apoptosis and the massive recruitment of neutrophils at the site of infection. The purified protein induces the permeability of pulmonary alveoli in mice, as well as severe pulmonary hypertension and dysfunction of the pulmonary barrier [38]. The leucocidin PVL of *S. aureus* indirectly induces deleterious effects on the lungs by increasing secretion of pro-inflammatory chemokines IL-8 and MCP-1 (monocyte chemotactic protein 1) by immune cells, which induces massive infiltration of monocytes at the site of infection. This infiltration is responsible for necrosis of lung tissue, alveolar hemorrhage, leading to rupture of the pulmonary barrier [39]. Thus, similar to ExlA, these PFTs, once delivered in the lung produce comparable deleterious effects, although using different mechanisms.

## 5. Caspase-1 Dependent Death of Macrophages

Pore formation by some PFTs induces additional effects on eukaryotic cells such as the activation of the inflammasome, ultimately leading to cell death [40]. ExlA-secreting bacteria induce inflammasome activation of primary macrophages by a two-step mechanism. The first, so called “priming” signal is mediated by the recognition of the LPS and the flagellum by TLR4 and TLR5, respectively, leading to transcription of genes encoding pro-inflammatory cytokines such as pro-Interleukin-1β (IL-1β). The second signal is initiated by the ExlA pore itself which leads to massive efflux of ions, among them potassium (K^+^). The decrease in K^+^ concentration triggers NLRP3-inflamasomme activation, which in turn activates caspase-1. Active caspase-1 induces the maturation of the pro-IL-1β into IL-1β. Caspase-1 also provokes the rupture of macrophage plasma membrane by a process called pyroptosis, releasing massive quantities of pro-inflammatory IL-1β in the surrounding environment [20] (Figure 2B). Therefore, similarly to T3SS-positive strains, ExlA-secreting bacteria are capable of triggering inflammasome activation and pro-inflammatory cell death of macrophages in vitro. How those effects contribute to overall pathogenicity of ExlA strains in vivo stays to be examined.

## 6. Exolysin Targets Host Cell Junctions

ExlA induces the degradation of E- and VE-cadherins, two adhesive proteins required for tissue integrity [32]. The cleavage is triggered by the ExlA pores formed in the plasma membrane and occurs earlier than plasma membrane breach. Following pore formation, a massive, rapid entry of calcium ions into the cytosol results in the maturation and activation of ADAM10, a eukaryotic protease inducing cadherin shedding (Figure 2C). In normal conditions, the ADAM10 precursor is maintained inactive within a complex with calmodulin, a protein having a high affinity for calcium. When intracellular calcium increases, calmodulin binds to calcium and dissociates from ADAM10, which in turn is proteolytically activated by furin, a proprotein convertase [41,42,43,44]. ADAM10 is then exported to the membrane, where it can exert its proteolytic activity against E- and VE-cadherins. Hence, bacterial strains secreting ExlA divert an endogenous cellular mechanism to compromise tissue integrity [32]. The disruption of adherens junctions by pore-forming toxins via ADAM10 activation was previously described for Hla from *S. aureus*, PLY from *Streptococcus pneumoniae* [45] and ShlA from *S. marcescens* [32]. While ADAM10 is the cellular receptor of Hla, two other toxins (PLY and ShlA) activate ADAM10 through pore-formation and calcium influx without using ADAM10 as the receptor [45,46], similar to ExlA. The dissociation of the junctions and the rupture of the plasma membrane induced by ExlA give an explanation for the striking effects observed in mouse alveoli during infection. This explains the facility of the bacteria to transmigrate across the lung mucosa and to colonize other organs.

## 7. Structural Features of Exolysin

Nothing is known about the molecular basis of the pore-forming activity of ShlA/ExlA toxins. In the case of ShlA, it has been reported that the hemolytic activity was virtually lost upon C-terminal truncation [47]. The C-terminal location of the activity is common in TPS substrates [48] and consistently, ExlA-mediated-hemolysis and cytotoxicity have been impaired when the last 295 residues of the protein were deleted [21]. PFTs belong to six classes clustered in two groups, α-helical and β-stranded, based on the secondary structure of the peptide lining the pore [33], with each family displaying characteristic stoichiometry and pore size. Due to the lack of homology to any other toxin and considering the lack of experimental data on pore formation or pore-forming (C-terminal) domain structure in the ShlA/ExlA family, how ExlA pores assemble in the host membranes is still unknown.

**Figure 3:**
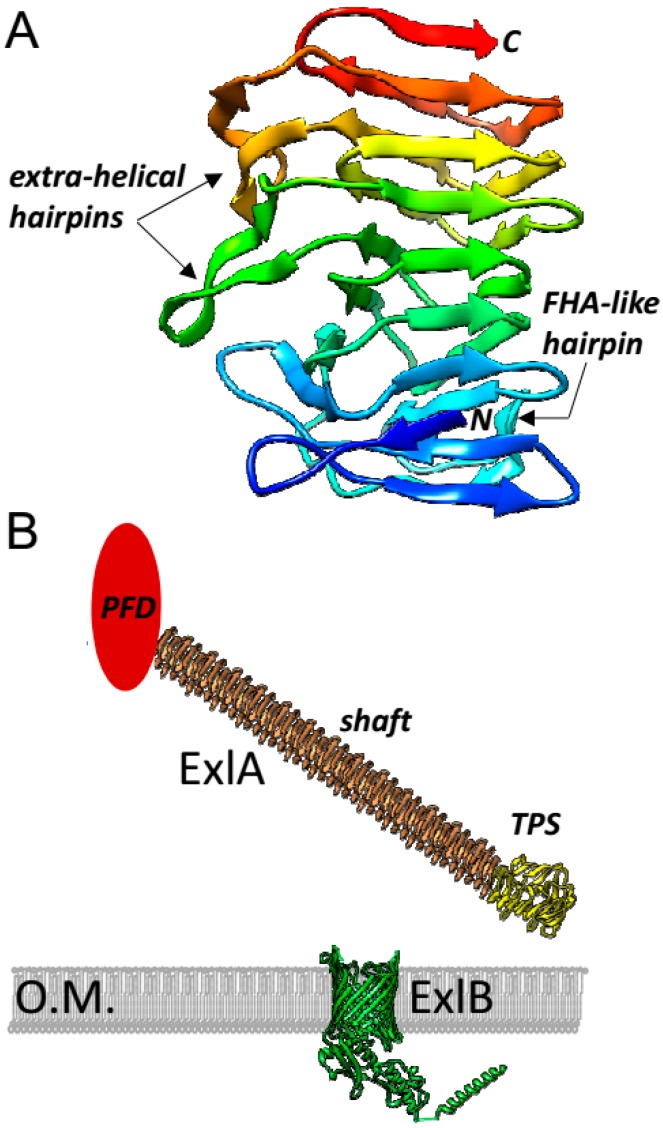
(**A**) Ribbon representation of the TPS domain of ExlA, as modeled by homology with HpmA as template by the Phyre2 webserver [53]. The polypeptidic chain is rainbow colored from the N- (blue) to the C-terminus (red). At its N-terminus, the helix is capped, possibly primed by three β-strands whose particular arrangement is conserved (a β-arc ending in a hairpin, here in blue). In FHA-like TPS domains, a loop is inserted in rung number 2 of the helix and is predicted to fold as a hairpin back onto the core, next to the cap [54]. In all TPS domains, two extra-helical elements stem from rungs number 4 and 6: in FHA and HpmA, hence probably in ExlA as well, these are hairpin structures folded next to each other onto the core [54,55]. (**B**) Model representation of ExlA and ExlB as they could be pictured from the X-ray structures of HpmA (Protein Data Bank code: 4W8Q) and FhaC (4QKY), respectively [54,55]. The filamentous domain of ExlA was modeled by replicating the three last rungs present in the structure of the HpmA TPS domain, after extra-helical hairpin trimming. To estimate the number of rungs in the shaft domain, the number of residues actually spanned by the Fil_Haemagg_2 repeats annotated in Pfam [25] (Figure 1A, same color code applies here) was divided by the average 20-residue length of a β-helical rung [56]. This allows to suggest that the shaft domain of ExlA contains approximately 41 rungs. As an average pitch of 4.8 Å was measured in β-helices of known structure, the shaft domain could be 200 Å-long, in addition to the 30 Å-length of TPS domains. The C-terminal domain of ExlA is displayed as an oval shape where a 30-kDa globular protein could fit, as no other structural information is available. The ribbon representation of the models was prepared with UCSF Chimera [57].

The C-terminal domain set aside, the structure of ShlA/ExlA proteins can be pictured by homology. TPS domains are very well conserved in sequence and in structure; sequence alignments allow to discriminate between at least two groups of TPS domains, depending on whether their closest homolog is *B. pertussis* FhaB, which is the case of ExlA, or *H. influenzae* HMW1A [48]. Several TPS domains have been crystallised to high resolution, including that of *P. mirabilis* HpmA which shares 44% identity with ExlA’s [29]. All TPS domains are built on a right-handed β-helix, i.e., a helical arrangement of β-strands where each rung of the helix is made of three β-strands and each strand contributes to one of the three parallel β-sheets that together make the helix (Figure 3A). Based on the structure of a complete TPS substrate [49], it is possible to envision how the helix primed in the TPS domain extends into the filamentous repeats-rich domain of ExlA to yield a fiber-like structure of 1000–1200 residues called the shaft domain (Figure 3B). A remarkable feature of ExlA is to display five or six Arg-Gly-Asp (RGD) motifs depending on the strain; these motifs are usually involved in cell-to-cell interactions through integrin binding [50]. All but one of the RGD motifs belong to the shaft domain (Figure 1A) and none displays a clear conservation pattern beyond *P. aeruginosa*. When all five RGD motifs were mutated to RGA in ExlA, the cytotoxicity of ExlA was preserved, implying that ExlA uses other ways to interact with eukaryotic cells [21]. Similarly, the filamentous hemagglutinin FhaB harbours an RGD motif whose mutation had no phenotype in vivo [51,52]. Thus, until now no clear function is specifically associated to the shaft domain. When pore formation was tested in vitro, full-length ExlA induced liposome leakage and C-terminal truncation was found to inactivate ExlA thus suggesting that the shaft domain is inactive [21]. Accordingly, the isolated C-terminal domain was also active but to a lower extent than the full-length ExlA. Therefore, the shaft may contribute indirectly to the function of the C-terminal domain, e.g., by supporting target cell binding, ExlA oligomerization and/or conformational changes of the C-terminus [21]. Altogether, the secretion of mature ExlA by *P. aeruginosa* would release the ~30 kDa cytotoxicity domain at the tip of a-more-than-230 Å-long fiber (Figure 3B).

## 8. Conclusions and Future Directions

ExlA is a toxin secreted by a growing family of *P. aeruginosa* strains, provoking devastating injuries in infected lungs. As such, ExlA-related histological lesions are highly different from those produced by classical *P. aeruginosa* strains, and patients infected with *exlA*+ strains should probably be treated differently than those infected with classical *P. aeruginosa* strains. Hospitals are not prepared to identify these types of strains. Relevant tests should be developed to specifically identify strains secreting Exolysin in patients, which would also help to estimate the incidence of these strains in hospital-acquired infections.

ExlA is a member of a distinct family of PFTs for which little structural information is available. One of the main hindrance to study its 3-dimensional structure and the formation of the pore within membranes is the instability of the toxin in solution, complicating its manipulation. The related toxin ShlA was previously purified [58], which brings hope for a possible purification of active ExlA in the future.

The expression of ExlA is variable in different strains, with some strains harboring the genes without any detectable protein secretion. These observations suggest that *exlBA* expression and/or ExlBA secretion are regulated by yet unknown mechanisms. As most virulence factors of *P. aeruginosa*, *exlBA* expression may be susceptible to complex regulatory circuits.

Two main cellular effects have been reported for ExlA: cadherin degradation via ADAM10 activation and induction of cell death in various cell types. The mechanisms of ExlA-dependent cell death in macrophages were clarified, yet they are unknown and likely different in other cell types, including epithelial and endothelial cells. It will also be of particular importance to understand why membrane repair mechanisms, induced by several other PFTs [59,60,61,62], are ineffective for ExlA.

Finally, the identity and the nature of the cellular receptor for ExlA are unknown. The receptor is probably quite ubiquitous as many cell types are susceptible to ExlA toxicity. Large screening approaches or biochemical copurification studies should be undertaken to identify the host receptor(s) of ExlA.

The history of the ExlA toxin started in 2014 [19] and is still at its very beginning. The next few years should bring more information about the properties of the protein and more details on its mode of action, information that is needed to eventually prevent its action.
